# Effects of simultaneous aerobic and inspiratory muscle training on diaphragm function, respiratory muscle strength, endurance, and fatigue index: randomized-controlled trial

**DOI:** 10.1007/s00421-025-05868-1

**Published:** 2025-07-02

**Authors:** Zeliha Çelik, Nevin A. Güzel, Seriyye Allahverdiyeva, Halit Nahit Şendur, Mahi Nur Cerit

**Affiliations:** 1 Department of Physiotherapy and Rehabilitation, Faculty of Health Sciences, Amasya University, Amasya, Türkiye; 2 Department of Physiotherapy and Rehabilitation, Faculty of Health Sciences, Gazi University, Ankara, Türkiye; 3 Department of Radiology, Saf Hospital, Baku, Azerbaijan; 4 Department of Radiology, Faculty of Medicine, Gazi University, Ankara, Türkiye

**Keywords:** Inspiratory muscle training, Aerobic exercise, Diaphragm, Pulmonary function tests, Athletic performance

## Abstract

**Purpose:**

This study aims to investigate the effects of Walking-Specific Inspiratory Muscle Training (W-SIMT) on diaphragm thickness–stiffness, pulmonary and respiratory muscle functions, and fatigue index.

Research question.

Do individuals who performed W-SIMT have better diaphragm functions, respiratory muscle strength, endurance, and performance?

**Methods:**

Twenty-seven healthy participants were included in the randomized-controlled study. The simultaneous inspiratory and aerobic exercise training (60–80% of maximal heart rate; 3 days/weeks; 4 weeks) was performed in the W-SIMT group (*n* = 14). The same aerobic (walking) exercise training protocol (*n* = 13) without Inspiratory Muscle Training (IMT) was applied in the walking (aerobic) group. The W-SIMT group performed 30 consecutive inspirations with 2-min rest (2 sets) in 50% of Maximal Inspiratory Pressure (MIP) using an assistive inspiratory muscle training device simultaneously while walking. The diaphragm thickness and stiffness, pulmonary functions, MIP, Maximal Expiratory Pressure (MEP), sustained inspiratory maximal pressure (P_max_), and fatigue index were evaluated before and 4 weeks after the intervention.

**Results:**

There was a significant effect of group × time interaction for MIP (cmH₂O and %, respectively) (F = 28.719, *p* < 0.001; F = 10.640, *p* = 0.003), sum (Pmax) (F = 5.414, *p* = 0.029), and fatigue index (F = 4.775, *p* = 0.039), in favor of the W-SIMT group.

**Conclusion:**

The distinctive combination of respiratory muscle training and aerobic exercise shows additional benefits enhancing particularly respiratory and anaerobic performance even over a short period. Further studies are needed to explore the long-term effects of W-SIMT.

**Trial Registry:**

Trial Registration Number: NCT06742372.

## Introduction

Respiratory muscle fatigue leads to the accumulation of metabolites like lactic acid and triggers reflex sympathetic activation via group III and IV nerve afferents, causing vasoconstriction in the extremities and locomotor muscle fatigue during exercise. Evidence emphasizes that respiratory muscle training reduces respiratory muscle fatigue by reducing or delaying the aforementioned metaboreflex mechanism, consequently improving performance (Chan et al. 2023; Hill 2000). The studies demonstrate that inspiratory muscle training effectively improves inspiratory muscle performance, including strength and endurance, and exercise capacity in patients with pulmonary conditions and athletes (Lötters et al. 2002; de Sousa et al. 2021). Inspiratory muscle strength training is applied with different intensities, durations, and repetitions. Inspiratory Muscle Training (IMT) performed at moderate load increases muscular hypertrophy and strength by improving peak inspiratory flow rate and maximal power (Romer and McConnell 2003). Previous studies emphasize that 50% and above inspiratory workloads provide greater strength and functional gains (Enright and Unnithan 2011; Rodrigues et al. 2018). On the other hand, aerobic exercise also contributes to functional performance by increasing oxidative capacity, costal diaphragm, and locomotor muscles (Powers and Criswell 1996). Previous studies have examined the effects of inspiratory muscle training (IMT) or aerobic exercise independently (Winkelmann et al. 2009; Liu et al. 2021), with limited data available on their concurrent application. Some research has suggested that combining IMT with aerobic exercise may provide functional benefits including improvements in respiratory muscle function (Matos et al. 2020; Hellyer et al. 2015). Matos et al. investigated the effects of simultaneous IMT and Nordic walking and proved that the combined training has positive effects on lung function and quality of life (Matos et al. 2020). Hellyer et al. found greater respiratory muscle activation during simultaneous stationary cycling and IMT (Hellyer et al. 2015). Although these findings are present, there is still limited evidence on whether simultaneous inspiratory muscle training and aerobic exercise provide improvements in respiratory and functional outcomes compared to aerobic training alone. The varying protocols and study design limit the comparability of the results obtained. Considering the interaction between respiratory and locomotor muscles, concurrent training may enhance functional benefits, and the inclusion of imaging-based evaluations may provide a novel and valuable approach to understanding the physiological effects of IMT. The potential physiological effects of the concurrent training should be further investigated, particularly with regard to its role in improving functional outcomes in individuals. Therefore, the study aims to investigate the effects of Walking-Specific Inspiratory Muscle Training (W-SIMT) on diaphragm thickness and stiffness, pulmonary functions, respiratory muscle strength and endurance, fatigue index, and body composition by combining both functional and imaging-based outcomes. The study tested the hypothesis that individuals who performed W-SIMT would have better diaphragm function, respiratory muscle function, and performance compared to those in the Walking (W) (aerobic) group.

## Materials and methods

### Study design and participants

The randomized-controlled and prospective study was approved (No: E-77082166-604.01.02-310783) by the Gazi University Ethics Committee and performed in compliance with the Declaration of Helsinki. All individuals signed an informed consent form before inclusion of the study. Healthy individuals who met the participation criteria and applied via the announcement board in university-affiliated clinics were randomized according to the order of application. The trial registration number was obtained. The participants were randomly allocated to either the W-SIMT or the W (aerobic) group using a computer-generated simple randomization method via an online randomization program (https://www.studyrandomizer.com) (Randomizer program, Software Application). The allocation was conducted based on a pre-generated randomization list, ensuring a completely random assignment to maintain allocation concealment and minimize selection bias. Individuals aged between 18 and 40 years who have physical activity levels above 600 MET-min/week according to the International Physical Activity Questionnaire (Lee et al. 2011) were included in the study. Individuals who were diagnosed with any cardiopulmonary or other chronic diseases, have any infections or limitations preventing them from doing an activity during assessment or intervention, do exercise regularly, and have smoked over 10 pack-years were excluded from the study. The radiologist performing the evaluation and the researcher conducting the analysis were blinded to the group allocation of the participants.

### Interventions

Exercise training and evaluations, except for diaphragm thickness and stiffness, were carried out by physiotherapists. The assessment of diaphragm function was performed by experienced radiologists. The same experts conducted the first and second evaluations to avoid differences between the measurements of the same individuals. All individuals' exercise programs were applied under the supervision of physiotherapists. All outcome measures were performed in the same order for each participant to ensure consistency. The evaluation sessions were divided into 2 days. Diaphragm thickness and stiffness (first day), pulmonary functions (first day), respiratory muscle strength and endurance (second day), and anaerobic performance (second day) were evaluated. There was a 1-hour rest period between the respiratory muscle performance tests including strength and endurance, and the anaerobic performance test. The second assessments were conducted 4 weeks later at the same time of day as the initial evaluations.

**Fig. 1:**
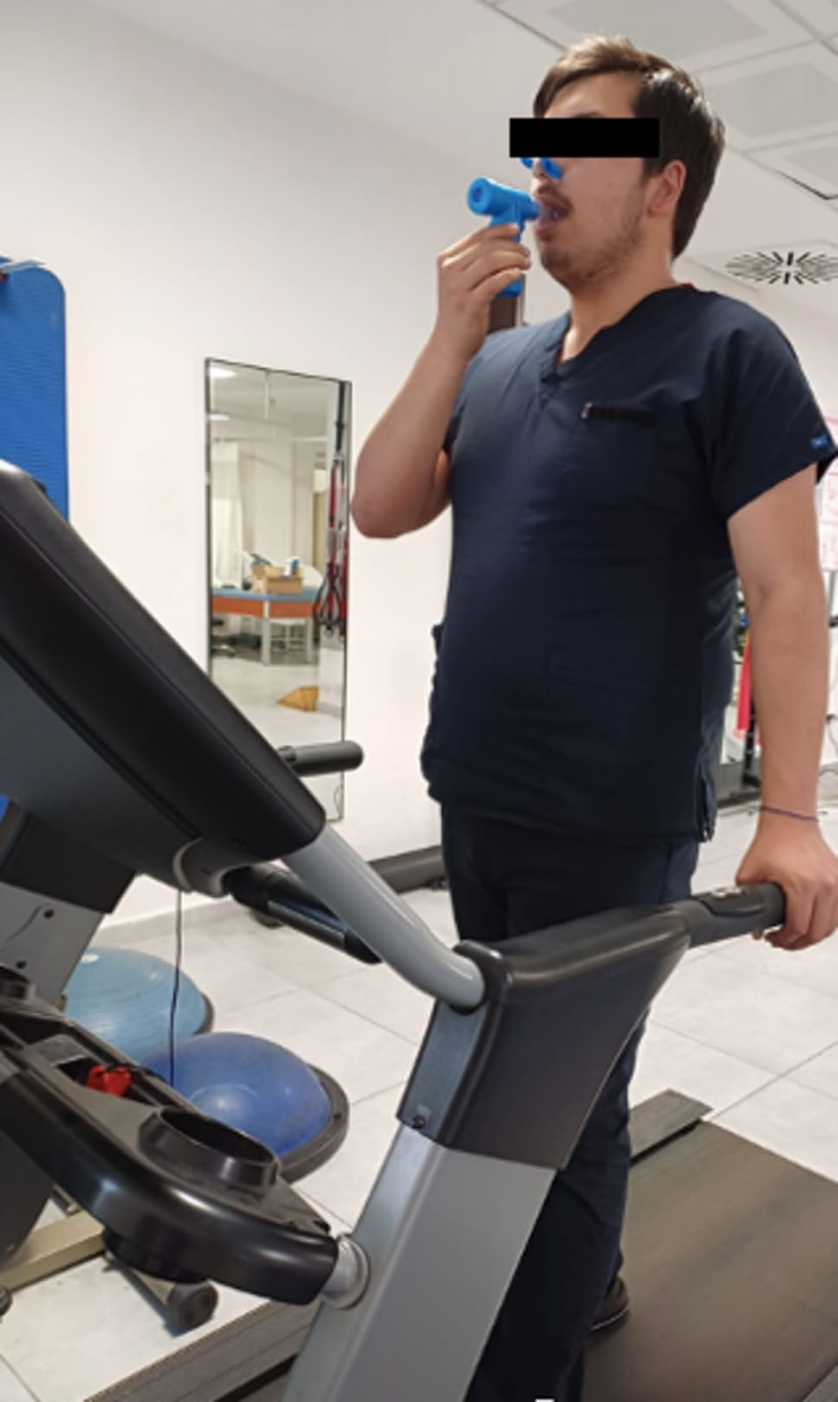
Illustration of the walking-specific inspiratory muscle training (W-SIMT) protocol. After a 5-min warm-up on the treadmill, participants performed 30 consecutive inspiratory efforts using an inspiratory muscle training device at 50% of their Maximal Inspiratory Pressure (MIP) while walking. This was followed by 2 min of walking without the device for recovery. After repeating this cycle once more, the exercise continued with the remaining time of the 20-min main walking session and concluded with a 5-min cool-down period. Heart rate, dyspnea, and fatigue levels were monitored throughout the session

The aerobic exercise training protocol (walking on a treadmill) was the same for both groups. In the W (aerobic) group, the training included walking on a treadmill without IMT. In the W-SIMT group, aerobic exercise training (walking on a treadmill) consisted of a 5-min warm-up, a 20-min loading period at 60–80% of maximal heart rate, and a 5-min cool-down. Simultaneously, IMT was integrated into the main exercise period. After completing the 5-min warm-up while walking, participants performed IMT at 50% of their Maximal Inspiratory Pressure (MIP) using an inspiratory muscle training device (PowerBreathe, HaB International Ltd., Southam, UK). Specifically, while walking on the treadmill, they executed 30 consecutive inspiratory efforts using the device, followed by 2 min of walking without IMT to rest. This cycle (30 breaths with the device while walking + 2-min walking) was repeated once more (Fig. 1). After the second cycle, participants continued walking without IMT for the remainder of the main exercise period. The MIP was measured every week, and the new inspiratory workload (50% of MIP) was adjusted.

During all exercise sessions, heart rate, perceived general fatigue and upper leg fatigue were observed by physiotherapists in both groups. Before and after training, vital signs, including heart rate, oxygen saturation, and blood pressure, were recorded. The type, intensity, duration, and frequency details of aerobic exercise training and IMT are given in Table 1.

**Table 1:** The training protocols of groups

Training protocol	W-SIMT group	W (Aerobic) group
Aerobic exercise training	*Type:* Continuous aerobic exercise training (walking on a treadmill) *Duration:* 30 min *Intensity:* 60–80% of maximal heart rate; intensity of dyspnea, MBS: 4–6 points; general and upper leg fatigue, MBS: 5–7 points *Frequency:* 1 session/day, 3 days/week	*Type:* Continuous aerobic exercise training (walking on a treadmill) *Duration:* 30 min *Intensity:* 60–80% of maximal heart rate; intensity of dyspnea, MBS: 4–6 points; general and upper leg fatigue, MBS: 5–7 points *Frequency:* 1 session/day, 3 days/week
Simultaneous inspiratory muscle training	*Type:* Inspiratory Muscle Training (IMT) (PowerBreathe, HaB International Ltd., Southam, UK) *Intensity: 5*0% of MIP (30 inspiration-2-min resting/set; 2 sets); after warm-up during walking *Frequency:* 1 session/day, 3 days/week	

*MBS* Modified Borg Scale, *min* minute

### Pulmonary functions and respiratory muscle strength

The pulmonary functions including Forced Vital Capacity (FVC), Forced Expiratory Volume in First Second (FEV_1_), Forced Expiratory Volume in First Second/Forced Vital Capacity (FEV_1_/FVC), Peak Expiratory Flow (PEF), Forced Expiratory Flow From 25 to 75% FEF_2575_ (%), MIP, and Maximal Expiratory Pressure (MEP) were evaluated with spirometer (Pony FX, COSMED Inc., Italy) and expressed as percentages of the predicted values. The assessments were performed according to American Thoracic Society/European Respiratory Society (ATS/ERS) guidelines (European Respiratory Society and American Thoracic Society 2002). At least three and five successful measurements were applied for dynamic lung volume and MIP-MEP values, respectively, and the highest value was selected for analysis (Quanjer et al. 1993). Reference equations adjusted according to age and body mass index were used to calculate the percentages of MIP–MEP values (Sanchez et al. 2018).

### Inspiratory muscle endurance

Inspiratory muscle endurance was evaluated with an inspiratory muscle training device (PowerBreathe, HaB International Ltd., Southam, UK). An incremental threshold loading test was performed starting from 30% (level 1) of MIP values in all participants. The test was continued by increasing the pressure by 10% every two minutes until participants reached 100% (level 8) of the MIP values. The new resistance adjustments were made without removing the device from the individual's mouth. The test was stopped in case of extreme fatigue, dyspnea, and inability to take two consecutive deep breaths. Sustained inspiratory maximal pressure (P_max_), time (T_max_), and reached level (Level_max_) were recorded. Breathing duration for less than 1 min in test steps was not included in the T_max_ calculation (Woszezenki et al. 2017).

### Diaphragm thickness and stiffness

**Fig. 2:**
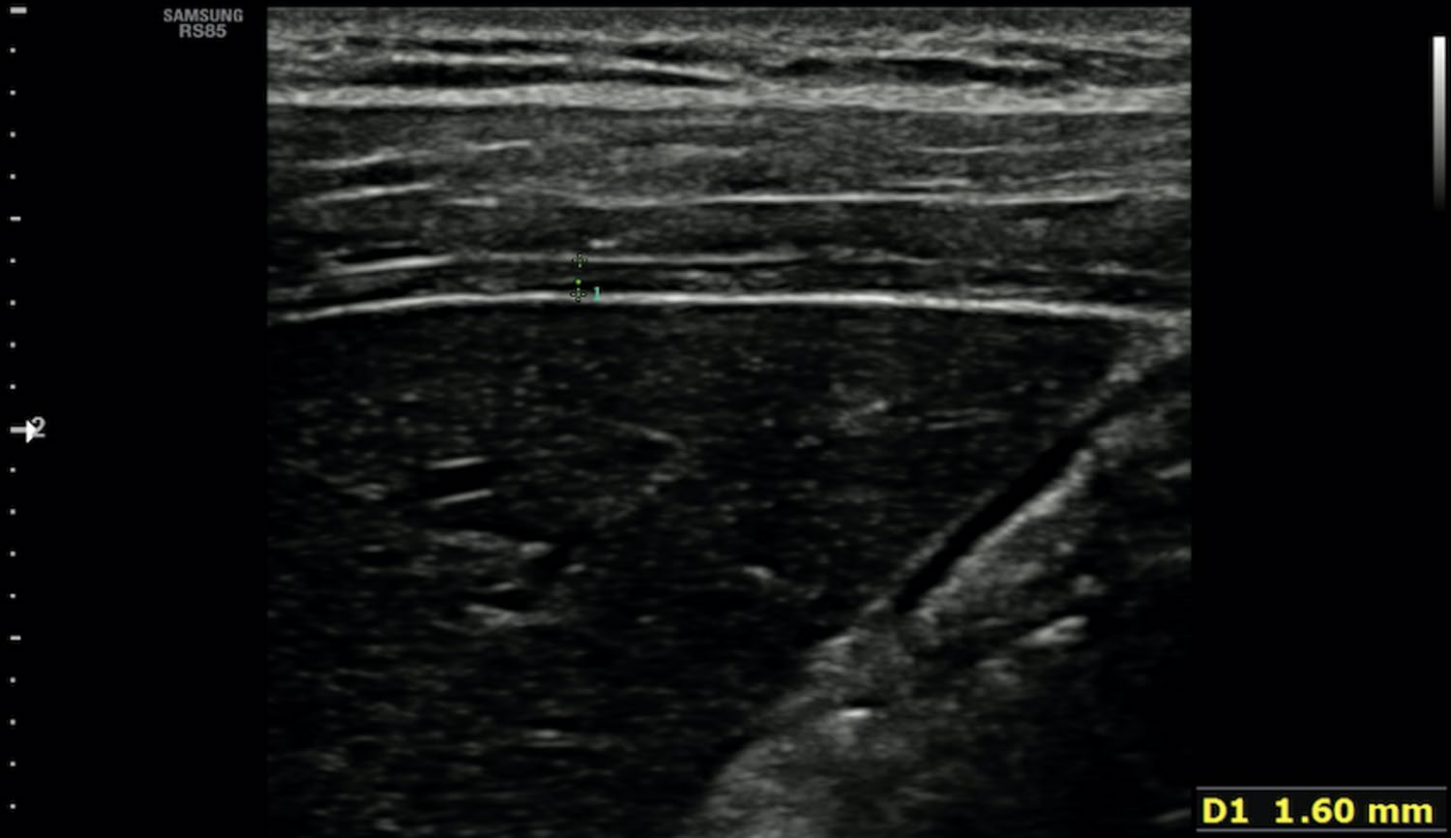
The evaluation of thickness of the diaphragm (gray-scale US image). Diaphragm thickness was measured during breath-hold at both inspiration and expiration phases

**Fig. 3:**
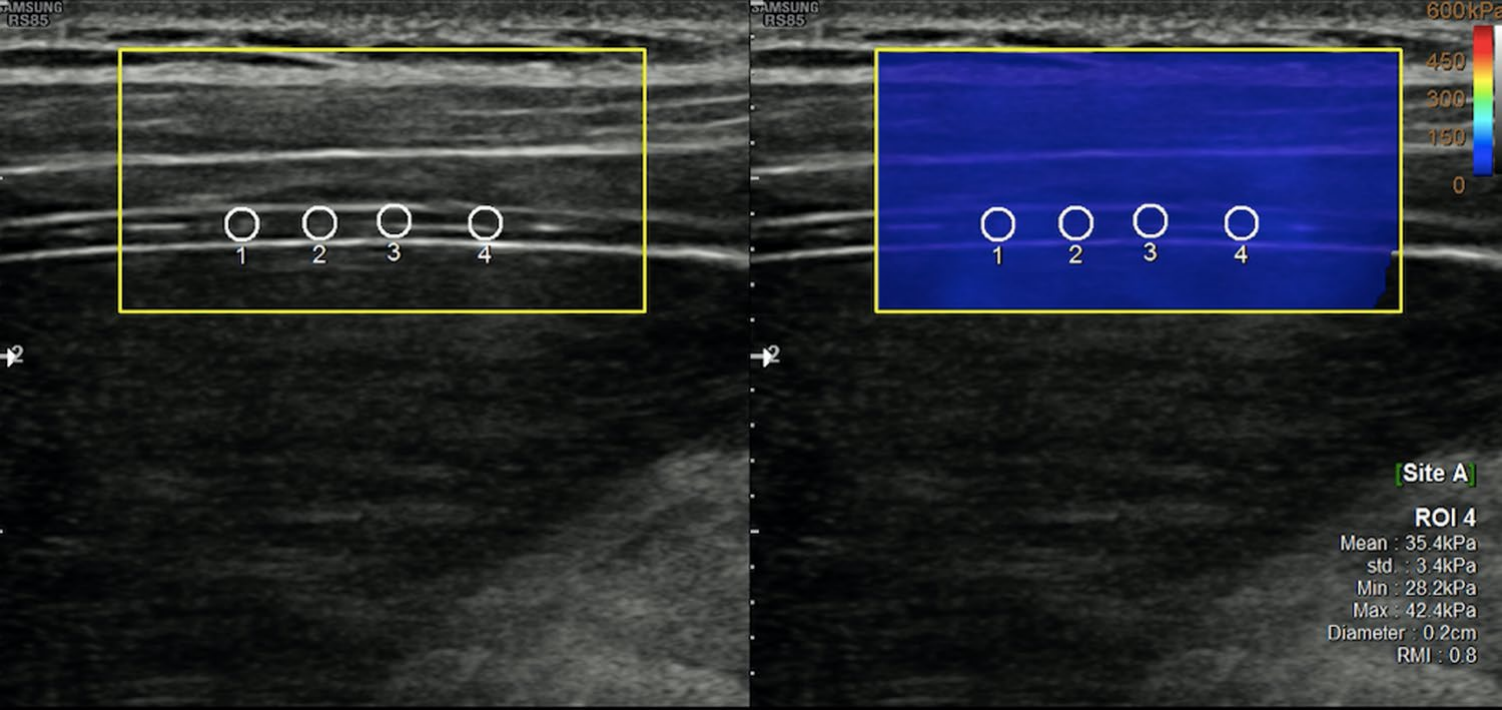
The evaluation of stiffness of the diaphragm. Repeated stiffness measurements for each respiratory phase (inspiration and expiration) were evaluated using shear-wave elastography (SWE) imaging

Diaphragm thickness and stiffness were evaluated using a single ultrasound device (RS 85, Samsung) with linear transducer (Fig. 2; Fig. 3). Right hemidiaphragm of all participants was evaluated in prone position, while the right hands were positioned above the head. At the ninth or tenth intercostal space at the midaxillary level, thickness and elasticity measurements were obtained, while the probe was held perpendicular to the chest wall. The observer paid particular attention not to apply any pressure with a transducer. The thickness of the diaphragm was measured three times during the breath-hold situation of both the inspiration and expiration phases. The median value of three measurements for each respiratory phase was noted for analyses. Just after the thickness measurements, Shear Wave Elastography (SWE) imaging was performed. For each case, 4 ROIs of approximately 2 mm in size were placed on the muscular layer of the diaphragm, and the mean of the 4 ROIs was noted. For each respiratory phase, these stiffness measurements were repeated 3 times, and median values were noted for analyses.

### Anaerobic performance

To verify the physiological effectiveness of the walking training protocol, performance was assessed with Running-Based Anaerobic Sprint Test (RAST). The RAST was performed to assess fatigue index and average power. The RAST consists of six maximal sprints (35 m) and a 10-s rest period at the end of each sprint. The participants’ body weight and six sprint times were used for calculation. Power was calculated for each sprint (power = (body mass × distance^2^)/time^3^), and the average power value was found by taking the average of six power values. Fatigue index was calculated with the [fatigue index% (FI = (peak power-minimum power/peak power) × 100)] formula. A high fatigue index value indicates a decrease in sprint performance (Zagatto et al. 2009).

### Body composition

Weight, fat mass, fat mass percentage, and body mass index were evaluated using TANITA bioelectrical impedance analysis (BC418-MA, TANITA Corporation, Tokyo, Japan) (Gallagher et al. 2000).

### Other assessments

The maximal heart rate was calculated according to the Eq. (208—0.7 × age) for healthy individuals (Tanaka et al. 2001). The heart rate using the heart rate monitor (Polar Electro Oy, Kempele, Finland) and the dyspnea, general and upper leg fatigue using the Modified Borg Scale, which has been widely used to evaluate fatigue and perceived exertion in various clinical and exercise settings (Borg 1982; Kendrick et al. 2000; Núñez-Cortés et al. 2022), were evaluated during assessments and training. Prior to the test, participants were introduced to the Modified Borg scale (Borg 1982) and instructed on how to report their perceived general fatigue and upper leg fatigue. Participants were specifically instructed to focus on the fatigue perceived in the front upper leg region (quadriceps muscle) and to distinguish this from the perception of general fatigue.

### Statistical analysis

According to the study of Liu et al. (Liu et al. 2021), the sample size estimation was calculated for total of 28 participants according to the respiratory muscle strength parameter (two-tailed; 80% power, α = 0.05, d = 0.55) using G*Power 3.0.10 system (Franz Faul, Universität Kiel, Germany) (Faul et al. 2007).

Data analysis was performed using the statistical software package SPSS version 20 statistical analysis program (2011, Version 20.0, IBM Corp., Armonk, NY). The Shapiro–Wilk test was used to assess the normality of the data. The means (X)-Standard Deviation (SD) and Median-Interquartile Range (IQR) were used for descriptive analyses of normally and non-normally distributed variables, respectively. The percentage (%) and frequency (n) were used to express categorical variables. The Student’s T, Mann–Whitney U, and Chi-square tests were used to analyze normally distributed, non-normally distributed, and categorical baseline values, respectively. Paired Sample T and Wilcoxon signed-rank tests were used to compare within the group before and after the evaluation. Group, time, and group × time interaction effects were evaluated using mixed-model analysis of variance (ANOVA). Logarithmic transformation was applied to non-normally distributed data prior to mixed-model ANOVA. Post hoc statistical power (1 – β) was calculated using the G-Power software (Faul et al. 2007) according to the MIP and diaphragm thickness. The statistical significance was defined as p < 0.05.

## Results

**Fig. 4:**
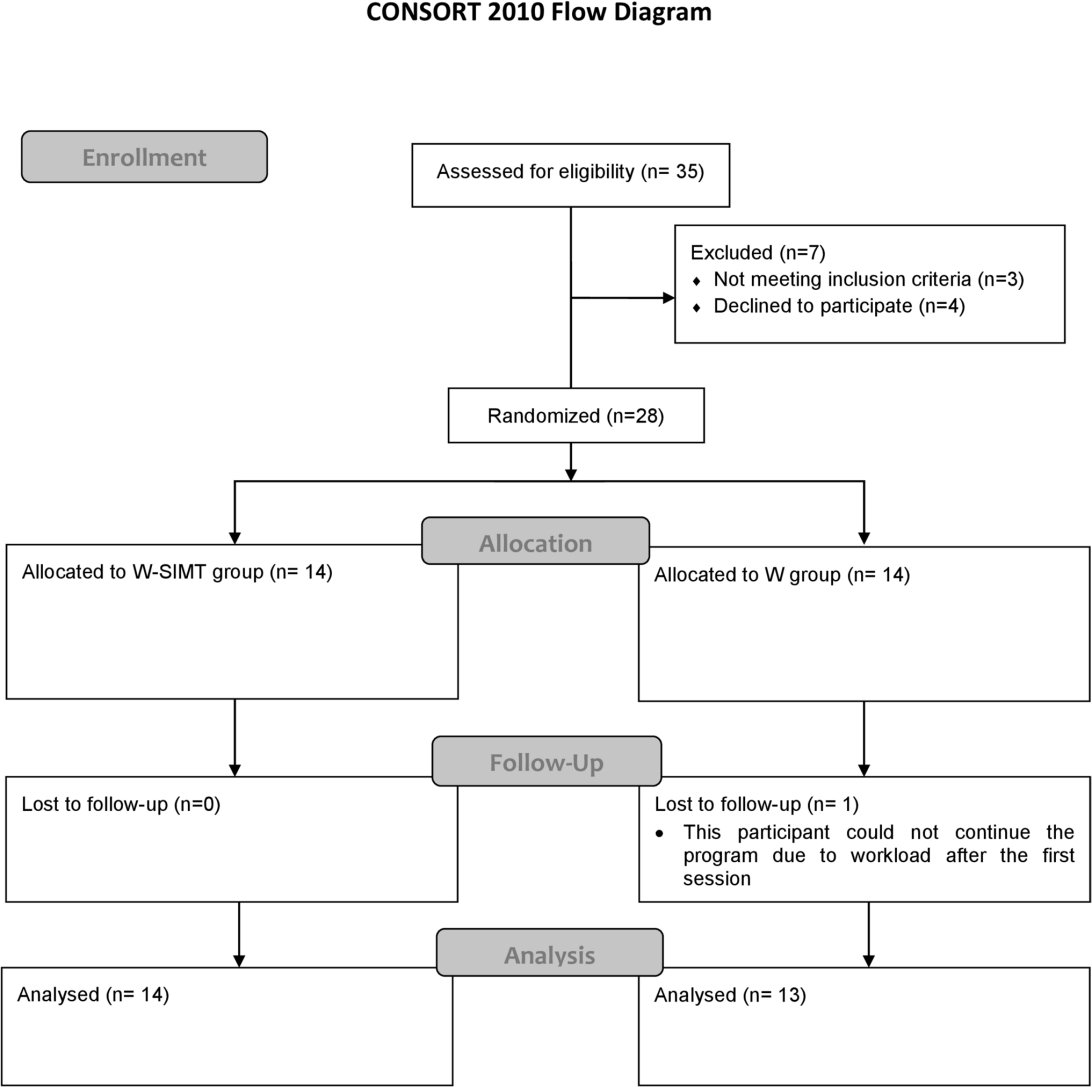
Flow diagram of participants. Explanation: CONSORT 2010 flow diagram illustrating the enrollment, allocation, follow-up, and analysis of participants in the study

The flow diagram is demonstrated in Fig. 4. Seven participants were excluded from the study. One participant had an allergic reaction before the assessment. The other two participants had influenza infections. The four participants gave up after learning the content of the training program. After randomization (*n* = 28), one participant dropped out of the study in the W (aerobic) group. Fourteen participants from the W-SIMT group and 13 from the W (aerobic) group were included for statistical analysis. All participants completed the program and did not experience any adverse events throughout the training program and assessments in both groups.

**Fig. 5:**
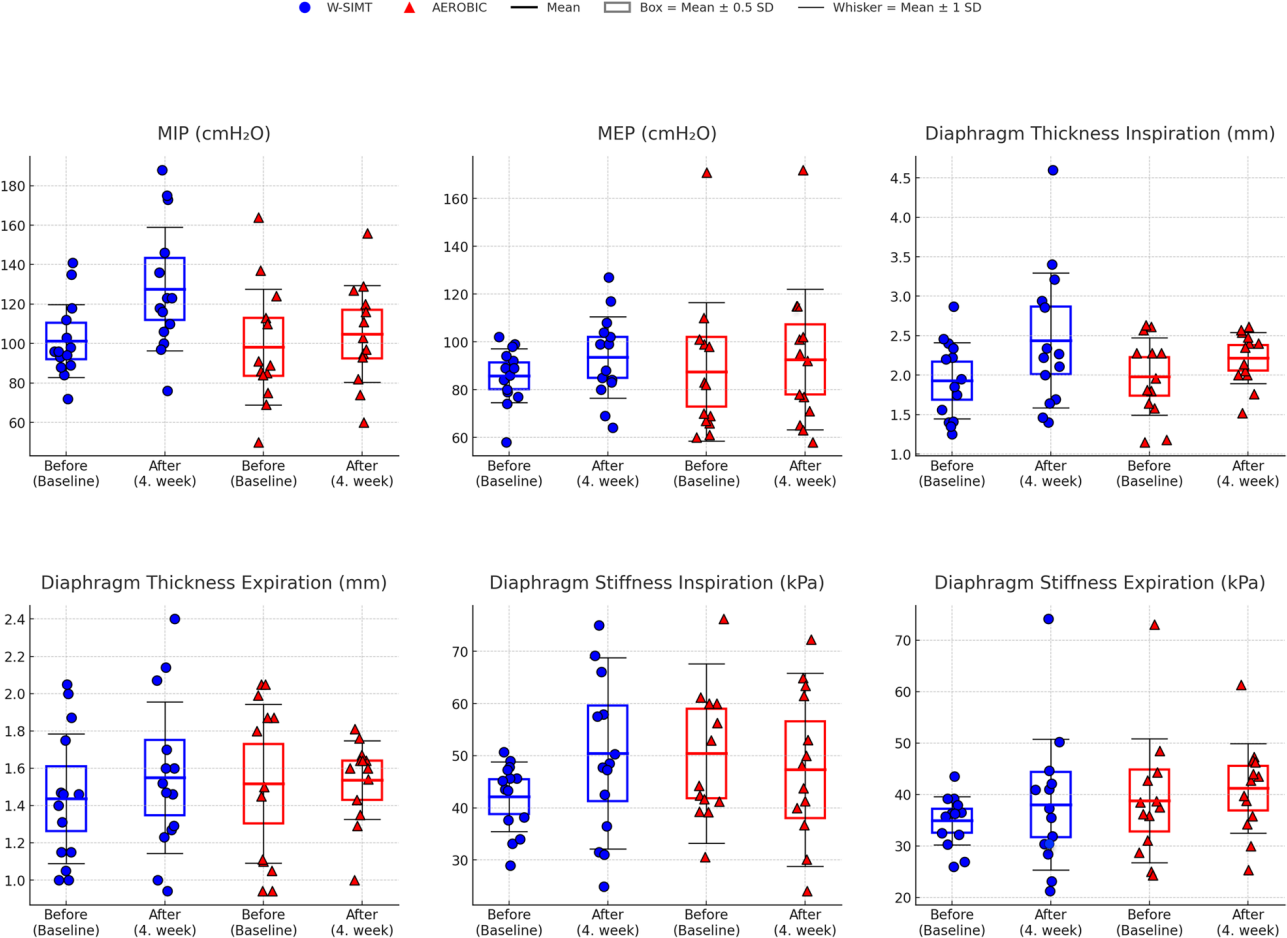
The changes in respiratory muscle performance and diaphragm functions in groups. Changes in maximal inspiratory pressure (MIP), maximal expiratory pressure (MEP), diaphragm thickness (during inspiration and expiration), and diaphragm stiffness (during inspiration and expiration) in the W-SIMT and AEROBIC groups before (Baseline) and after the 4-week intervention. The central horizontal line represents the mean value. The boxes indicate mean ± 0.5 standard deviation (SD), and the whiskers represent mean ± 1 SD

The baseline demographic (Table 2), respiratory (Table 3), performance, and anthropometric (Table 4) parameters were similar in groups (*p* > 0.05). The comparisons before and after the intervention within the group are shown in Table 3 and Table 4. The post hoc power (1 – β) was found to be 0.81 for diaphragm thickness during inspiration and 0.96 for MIP. The changes in respiratory muscle performance and diaphragm function in groups are presented in Fig. 5.

**Table 2:** The baseline characteristics of groups

Characteristics	W-SIMT group (*n* = 14) (X ± SD)	W (Aerobic) group (*n* = 13) (X ± SD)	*p*
Age, year	22.00 ± 3.01	22.69 ± 4.52	0.641
Female; Male, n/%	12/85.7%;2/14.3%	11/84.6%;2/15.4%	0.673
Weight, kg	57.65 ± 10.39	65.91 ± 14.29	0.097
Height, cm	166.36 ± 10.08	166.62 ± 7.36	0.940
BMI, kg/m^2^	20.83 ± 3.17	23.64 ± 4.37	0.080
Smoking (current; non-smoker, n/%)	1/7.1%;13/92.9%	1/7.7%;12/92.3%	0.367
IPAQ, MET-minutes/week	1041.00 ± 347.58	1000.25 ± 486.49	0.885

*IPAQ* International Physical Activity Questionnaire, *BMI* body mass index, *kg* kilogram, *cm* centimeter, *m* meter, *p < 0.05

The Student’s T and Chi-square tests were used to analyze normally distributed and categorical baseline values

W-SIMT Group, Walking-Specific Inspiratory Muscle Training Group

W (Aerobic) Group, Walking (Aerobic) Group

**Table 3:** Comparison of respiratory parameters within and between groups

		W-SIMT Group	W(Aerobic) Group	F	P*	ηp2	F	P*	ηp2	F	P*	ηp2
		(*n* = 14)	(*n* = 13)									
MIP (cmH_2_O)	Before	101.36 ± 19.18	98.23 ± 30.60	1.642	0.212	0.062	10.265	**0.004**	0.291	28.719	** < 0.001**	0.535
	After	127.64 ± 32.55	104.85 ± 25.61									
	p^µ^	** < 0.001**^**µ**^	0.054									
	^Baseline p§^	0.751										
MEP (cmH_2_O)	Before	85.79 ± 11.72	87.46 ± 30.20	0.002	0.964	0	7.114	**0.013**	0.222	0.282	0.6	0.011
	After	93.50 ± 17.72	92.62 ± 30.59									
	p^µ^	**0.036**^**µ**^	0.17									
	^Baseline p§^	0.854										
MIP (%)	Before	85.61 ± 13.21	79.12 ± 20.71	4.473	**0.045**	0.152	30.277	** < 0.001**	0.548	10.64	**0.003**	0.299
	After	107.55 ± 24.04	84.73 ± 16.74									
	p^µ^	** < 0.001**^**µ**^	**0.043**^**µ**^									
	^Baseline p§^	0.337										
MEP (%)	Before	86.75 ± 12.87	84.73 ± 21.61	0.225	0.64	0.009	6.782	**0.015**	0.213	0.284	0.599	0.011
	After	94.64 ± 19.32	89.94 ± 23.15									
	p^µ^	**0.037**^**µ**^	0.186									
	^Baseline p§^	0.774										
FVC (%)	Before	97.39 ± 7.15	98.31 ± 11.92	0.098	0.757	0.004	2.341	0.139	0.089	0.166	0.687	0.007
	After	95.92 ± 7.84	97.46 ± 12.68									
	p^µ^	0.275	0.311									
	^Baseline p§^	0.952										
FEV_1_ (%)	Before	99.39 ± 9.13	98.46 ± 13.32	0.006	0.939	0	2.556	0.123	0.096	2.033	0.167	0.078
	After	96.69 ± 8.07	98.31 ± 14.41									
	p^µ^	0.083	0.888									
	^Baseline p§^	0.762										
FEV_1_/FVC (%)	Before	104.85 ± 8.86	103.39 ± 7.62	0.047	0.83	0.002	0.004	0.952	0	1.317	0.262	0.052
	After	104.15 ± 9.55	104.15 ± 8.86									
	p^µ^	0.541	0.254									
	^Baseline p§^	0.708										
PEF (%)	Before	87.00± 9.44	87.46 ± 13.35	0.182	0.673	0.008	4.387	**0.047**	0.155	0.467	0.501	0.019
	After	89.46 ± 8.67	92.31 ± 11.27									
	p^µ^	0.222	0.123									
	^Baseline p§^	0.955										
FEF_2575_ (%)	Before	90.69 ± 20.16	89.39 ± 22.22	0.003	0.953	0	0.172	0.682	0.007	0.719	0.405	0.029
	After	88.00 ± 19.65	90.31 ± 26.40									
	p^µ^	0.425	0.743									
	^Baseline p§^	0.856										
Diaphragm thickness												
Inspiration, mm	Before	1.93 ± 0.50	1.98 ± 0.53	0.14	0.712	0.006	11.817	**0.002**	0.33	1.539	0.227	0.06
	After	2.44 ± 0.89	2.22 ± 0.35									
	p^µ^	**0.013**^**µ**^	0.058									
	^Baseline p§^	0.788										
Expiration, mm	Before	1.44 ± 0.36	1.52 ± 0.46	0.069	0.795	0.003	0.741	0.398	0.03	0.395	0.536	0.016
	After	1.55 ± 0.42	1.54 ± 0.23									
	p^µ^	0.294	0.877									
	^Baseline p§^	0.62										
Diaphragm stiffness												
Inspiration, kPa	Before	42.13 ± 6.65	50.43 ± 17.19	0.274	0.605	0.011	0.483	0.494	0.02	2.335	0.14	0.089
	After	50.43 ± 18.32	47.32 ± 18.53									
	p^µ^	0.099	0.612									
	^Baseline p§^	0.138										
Expiration, kPa	Before	34.91 ± 4.86	38.81 ± 13.10	1.568	0.223	0.061	1.054	0.315	0.042	0.002	0.961	0
	After	38.04 ± 13.20	41.65 ± 9.27									
	p^µ^	0.449	0.512									
	^Baseline p§^	0.311										
Respiratory muscle endurance												
P_max_, mmHg	Before	57.31 ± 22.88	50.46 ± 36.32	2.765	0.109	0.103	20.143	** < 0.001**	0.456	5.414	**0.029**	0.184
	After	87.39 ± 28.68	60.00 ± 24.41									
	p^µ^	**0.001**	0.134									
	^Baseline p§^	0.571										
T_max_, s	Before	325.0(229.0;600.0)	240.0(120.0;518.0)	2.794	0.108	0.104	12.593	**0.002**	0.344	0.212	0.649	0.009
	After	589.50(419.25;720.0)	360.0(287.50;540.0)									
	pγ	**0.033**^**µ**^	0.075									
	^Baseline pɸ^	0.39										
Level_max_	Before	3.0(2.0;5.0)	2.0(1.0;4.50)	3.293	0.082	0.121	15.594	**0.001**	0.394	0.122	0.73	0.005
	After	5.0(3.75;6.0)	3.0(2.50;4.50)									
	p^γ^	**0.035**^**µ**^	0.085									
	^Baseline pɸ^	0.243										

Bold values indicate statistically significant differences (*p* < 0.05)

*FEV*_*1*_, Forced expiratory volume in the first second; *FVC*, Forced vital capacity; *FEV*_*1*_*/FVC*, Forced expiratory volume in the first second/forced vital capacity; *PEF*, Peak expiratory flow; *FEF*_*25–25%*_*,* Forced expiratory flow from 25 to 75%; *MIP*, Maximal inspiratory pressure; *MEP*, Maximal expiratory pressure; P_max_, Sustained inspiratory maximal pressure; T_max_, Sustained maximal time; Level_max_, Maximal level; *cmH*_*2*_*O*, centimeter water; mm, millimeter*; kPa, kilopascal; mmHg, millimeter mercury; s*, second; ηp2: Partial eta squared

Group effect, time effect, and group × time interaction effect were evaluated using mixed-model ANOVA (p* < 0.05)

p^µ^ and p^γ^ *Comparisons of Within-Group Before and After Assessments (μ: Paired Samples t test; γ: Wilcoxon test)*

*(Baseline p) Baseline comparisons between groups (§: Student’s t test; ɸ: Mann–Whitney U test)*

*W-SIMT Group, Walking-Specific Inspiratory Muscle Training Group; W (Aerobic) Group, Walking (Aerobic) Group*

**Table 4:** Comparison of performance and anthropometric parameters within and between groups

		W-SIMT Group	W(Aerobic) Group	F	P	ηp2	F	P	ηp2	F	p	ηp2
		(*n* = 14)	(*n* = 13)									
Fat mass, kg	Before	13.36 ± 5.30	18.81 ± 9.14	3.216	0.086	0.123	0	1	0	0	1	0
	After	13.36 ± 5.55	18.81 ± 9.20									
	p^µ^	1	1									
	^Baseline p§^	0.085										
Fat mass, %	Before	23.43 ± 6.28	27.19 ± 9.34	1.414	0.246	0.058	0.001	0.976	0	0.007	0.936	0
	After	23.39 ± 6.46	27.21 ± 9.15									
	p^µ^	0.932	0.973									
	^Baseline p§^	0.253										
RAST												
Fatigue index, %	Before	50.72 ± 12.87	46.47 ± 9.09	0.004	0.949	0	3.787	0.064	0.141	4.775	**0.039**	0.172
	After	42.25 ± 10.90	46.96 ± 5.09									
	p^µ^	**0.031**^**µ**^	0.815									
	^Baseline p§^	0.98										
Average Power	Before	101.10(84.80;162.25)	99.70(91.45;156.25)	0.011	0.919	0	10.772	**0.003**	0.329	0.101	0.753	0.005
	After	108.25(102.48;178.35)	117.70(101.85;172.65)									
	p^γ^	0.099	0.05									
	^Baseline pɸ^	0.728										

Bold values indicate statistically significant differences (*p*< 0.05)

RAST Running-Based Anaerobic Sprint Test*; kg* kilogram,* W/s* watt/second;* min* minute

Group effect, time effect, and group × time interaction effect were evaluated using mixed-model ANOVA (p* < 0.05)

p^µ^ and p^γ^ *Comparisons of Within-Group Before and After Assessments (μ: Paired Samples t test; γ: Wilcoxon test)*

*(Baseline p) Baseline comparisons between groups (§: Student’s t test; ɸ: Mann–Whitney U test)*

*W-SIMT Group, Walking-Specific Inspiratory Muscle Training Group; W (Aerobic) Group, Walking (Aerobic) Group*

The mixed-model ANOVA showed a significant effect of group × time interaction (F = 28.719, p < 0.001; F = 10.640, P = 0.003) for MIP (cmH_2_O and %, respectively), P_max_ (F = 5.414, p = 0.029) and fatigue index (F = 4.775, p = 0.039), in favor of the W-SIMT group. MIP (cmH2O; %), MEP (cmH_2_O; %), PEF%, diaphragm thickness during inspiration, respiratory muscle endurance parameters (P_max_, T_max_, Level_max_), and average power revealed a significant time effect (p < 0.05; Table 3, Table 4).

## Discussion

The results of our study investigating the assistive combined exercise training on the functional structure and strength of the diaphragm, respiratory functions, anthropometric characteristics, and anaerobic performance in participants prove that simultaneous IMT added to aerobic exercise has a prominent impact on especially inspiratory muscle strength and endurance, and fatigue index compared to aerobic exercise training alone.

There are no studies in the literature investigating the effects of aerobic (walking) training, including simultaneous inspiratory muscle strength training on respiratory and athletic performance in healthy individuals. One study applied simultaneous IMT with exercise, and respiratory training was integrated with core and postural muscle exercises in patients (Ozsoy et al. 2021). Matos et al. (Matos et al. 2020) discussed the positive aspects of inspiratory muscle strength training added to Nordic Walking regarding lung function (especially FVC and MEP values) and quality of life in Parkinson's patients. Hellyer et al. (Hellyer et al. 2015) performed IMT during cycling in healthy adult cyclists and found greater diaphragm activity and training effects in combination with IMT and cycling compared with IMT alone. The findings of our study are consistent with the recent studies that have investigated the positive effects of combining exercise and IMT. Considering the results of our study and these studies, simultaneously applied IMT added to aerobic exercise provides remarkable health-related improvements in a short time in healthy individuals and patients.

Inspiratory muscle training contributes to physiological adaptation, increasing the diaphragm muscle's cross-sectional area (McCool et al. 1997; Fabero-Garrido et al. 2022). In addition, increased MIP values are positively correlated with diaphragm thickness (Lee et al. 2023). Enright et al. (Enright et al. 2006) found that 8-week supervised IMT improves diaphragm thickness in healthy individuals. Our study showed that both groups experienced improvements in diaphragm thickness during inspiration over time, regardless of group distribution. This improvement may be attributed to physiological adaptations related to general training in both groups. Although no significant group × time interaction was found, the W-SIMT group demonstrated higher relative increases in diaphragm thickness and inspiratory muscle strength. Specifically, the W-SIMT group showed a 20.9% increase in diaphragm thickness and 20.58% in MIP. Downey et al. emphasized that IMT performed during 4 weeks resulted in an 8–12% increase in diaphragm thickness and an average of 4% improvement in MIP in healthy individuals (Downey et al. 2007). Compared to this study, the enhanced outcomes in the W-SIMT group may be due to the complementary effects of IMT combined with aerobic exercise in our study. In addition, the increases observed in the W-SIMT group are consistent with the improvements reported in studies that applied inspiratory muscle strength training alone for 8 or 16 weeks (DePalo et al. 2004; Enright et al. 2006). The early improvements observed in our study suggest a potentially beneficial role of the combined intervention. More studies are needed to confirm these findings. On the other hand, it is important to emphasize that improvements in diaphragm thickness occurred in both study groups. The potential contribution of aerobic training alone to structural respiratory adaptations should not be underestimated. The previous study indicated that diaphragm hypertrophy is not the sole cause of the increase in inspiratory muscle strength. Enhanced accessory muscle function and neural adaptations, including improved coordination of synergistic muscles, may increase inspiratory muscle strength (McConnell 2011). There is a need to clarify the physiological and neurological mechanisms leading to the development resulting from the W-SIMT. Furthermore, although the increase in inspiratory muscle strength was greater in the W-SIMT group, the improvement in expiratory muscle strength over time in both groups is clinically meaningful. Exploring the effects of training specifically aimed at the expiratory muscles is important.

Assessment of stiffness for the diaphragm using SWE is a novel, useful, and non-invasive method. Furthermore, SWE, which has been related to transdiaphragmatic pressure, provides valuable information for assessing diaphragmatic function (Bachasson et al. 2019). In our study, the diaphragm stiffness at the end of inspiration was higher than the values at the end of expiration in both groups. Similar to our study, Şendur et al. found that the stiffness of the diaphragm increased more with inspiration in healthy individuals. They emphasized that this situation may be related to the higher stiffness values in the contracted muscles, since the diaphragm contracts during inspiration (Şendur et al. 2022). Previous studies found that stiffness values showed a correlation with some physiological parameters, such as body mass index and lung volume (Xu et al. 2021). Therefore, further studies are needed to clarify physiological factors that may influence diaphragm stiffness.

Respiratory muscle endurance is related to airway resistance. Previous findings emphasized that the changes in respiratory muscle function, including respiratory muscle strength and endurance parameters, were associated with pulmonary involvement (Vendrusculo et al. 2016). Therefore, the assessment of respiratory muscle endurance is essential, since it is linked to inspiratory muscle performance and diaphragm function. Previous studies using different methods to evaluate respiratory muscle endurance in patients, athletes, and non-athletes reported that respiratory muscle training improves respiratory muscle endurance (Gualdi et al. 2015; Weiner et al. 2003). Similarly, the maximal achieved pressure was considerably improved in the W-SIMT group in our study. Although the maximal achieved duration and level improved over time in both groups, walking training alone was not effective in improving achieved pressure in our study. This emphasizes that the positive effects of combined training have a more profound impact on respiratory muscle endurance than walking alone. The most important difference between our study and the mentioned studies is that the training duration and frequency are lower. The effects of the specific inspiratory muscle strength training that individuals apply while walking without spending a separate training period are quite noticeable. Considering that athletes with good respiratory muscle function can perform better (Akınoğlu et al. 2019), the specific IMT added to training in sports branches may effectively achieve better performance in a short time. On the other hand, our results will guide future studies on the healing effects of these approaches in healthy individuals and individuals with chronic lung disease. Further studies are needed to determine appropriate protocols, including frequency and duration in different sports branches. In addition, it is crucial to investigate the minimal effective training durations and frequencies to optimize performance outcomes.

After IMT, the amount of oxygen taken up by the muscles per unit of time increases due to the improvement in performance, and the lactate threshold is delayed (Fernández-Lázaro et al. 2021). This mechanism suggests that IMT not only improves respiratory function but also contributes to better overall athletic performance, including endurance and fatigue resistance. In our study, improved fatigue index values (50%-42%; pre–post-values, respectively) in the W-SIMT group may be explained by this mechanism. Fernandez-Lazaro et al. (Fernández-Lázaro et al. 2021) highlight that IMT should include athletes’ routine training due to the potential benefits of IMT. Our results support their findings by showing that combining IMT with aerobic exercise enhances endurance and reduces fatigue, both crucial for athletic performance. Training the diaphragm, which has a major role in core and pelvic stabilization as well as its essential function in respiration, in specific positions such as walking and cycling can enable athletes to perform better during the game thanks to the respiratory-postural stabilization cooperation. This collaboration may enable an increase in performance through the improvement of diaphragm function in activities that require aerobic endurance and core stabilization.

The effect of inspiratory muscle training on aerobic capacity, especially oxygen consumption, has been investigated in various populations. Evidence from studies showed that IMT leads to significant improvements in VO₂max in patients with chronic heart failure (Dall’Ago et al. 2006) and individuals recovering from COVID-19 (Chen et al. 2023). In contrast, the effect of IMT on VO₂max in well-trained athletes remains inconsistent. While Riganas et al. reported improvements in inspiratory muscle strength without meaningful gains in VO₂max (Riganas et al. 2008), Rożek-Piechura et al. demonstrated that high-intensity IMT yields more pronounced benefits in long-distance runners (Rożek-Piechura et al. 2020). These variable results are thought to reflect differences in baseline fitness and the oxygen cost of breathing. In addition, IMT is believed to reduce respiratory muscle fatigue and the oxygen demand of breathing, thereby sparing oxygen for locomotor muscles, accelerating oxygen uptake, and improving endurance (Smith et al. 2020; Hill 2000). However, the optimal IMT protocols (intensity, duration, and timing) remain unclear and likely depend on the target population and training status. Although there is no study in the literature directly comparable to our results that investigates the effects of combined training, a systematic review investigating the effects of IMT on athletic performance observed significant enhancements in the Maximum Oxygen Uptake (VO_2_max) following 6 weeks of IMT associated with the MIP ≥ 21.5% improvements (Fernández-Lázaro et al. 2021). Similarly, the increase of MIP% (21.9%) in the W-SIMT group may explain enhancements in performance in our study. Our results provide evidence that W-SIMT strengthens the inspiratory muscles and enhances performance. Future studies should focus on the tests of evaluating the effect of direct VO_2_max values on specific IMT.

## Conclusion

The assistive combined exercise training performed in our study brings a new perspective to the literature. The results of our study revealed that the concurrent specific IMT added to aerobic exercise training improves particularly inspiratory muscle strength, endurance, and anaerobic performance even in a short time. The combination of respiratory muscle training with aerobic exercises shows potential to support muscle adaptation, which may contribute to gradual improvements in respiratory efficiency. Specific IMT needs to be planned and implemented individually to create unique programs for athletes and patients.

### Strengths and limitations

This study is the first to examine the effects of W-SIMT effects on diaphragm thickness and stiffness, respiratory strength and endurance, and fatigue index. Individuals achieved better physiological and functional gains in a shorter time thanks to this combined training. Creating an alternative training program without wasting additional time for individuals with time constraints has provided a new perspective.

The limitation of this study is the short duration of the follow-up period, which limits the ability to evaluate the long-term effects of the training program on diaphragm structure, respiratory function, and anaerobic capacity. Further research with longer follow-up periods is needed to determine the persistent benefits of the W-SIMT. Another limitation is the absence of a dedicated IMT group in this study, which was designed to investigate the effects of the combination of aerobic exercise and IMT. Future research that isolates the effects of IMT alone and compares them with the potential additional benefits provided by the combination could provide valuable information. In addition, in our study, assessments based on oxygen consumption, which could have provided more objective results in evaluating aerobic capacity, could not be performed due to technical limitations.

## Data Availability

The data that support the findings of this study are available on request from the corresponding author.
